# Mental health care and delivery system at Temeke hospital in Dar es Salaam, Tanzania

**DOI:** 10.1186/s12888-017-1271-9

**Published:** 2017-03-23

**Authors:** Joel Seme Ambikile, Masunga K. Iseselo

**Affiliations:** Department of Clinical Nursing, Muhimbili University of Health & Allied Sciences (MUHAS), P.O. Box 65004, Dar es Salaam, Tanzania

**Keywords:** Mental health services, Mental health care, Challenges of mental health services, Meeting mental health needs

## Abstract

**Background:**

Low and middle income countries face many challenges in meeting mental health needs in their regions. Treatment of patients with mental disorders is inadequate in most of these countries and majority of individuals with severe mental disorders remain untreated. The bad news is that mental health problems in these countries are on the rise due to socioeconomic challenges being faced. Regardless of guidelines and directives provided by WHO for organizing mental health services, these countries continue to face many challenges in the effort to cater for mental health needs. Such challenges include lack of human resource for mental health especially at primary health care level, inadequate training of human resource for mental health, misplacement of human resource for mental health, lack of drugs, wrong priorities, problematic insurance coverage for mental disorders, and stigma. This study aimed at exploring mental health care and delivery system at Temeke district hospital, and how services were organized to meet the increasing mental health needs of its population.

**Methods:**

A qualitative study was conducted at Temeke hospital in Dar es Salaam, involving 7 in-depth interviews with mental health care providers, 7 in-depth interviews with mentally ill patients, and 2 focus group discussions with caregivers. A convenient sampling procedure was used to select participants for the study. All interviews were audio-recorded in Kiswahili and transcribed. A qualitative Content Analysis was used to analyze data after translation with the aid of Nvivo 10 software.

**Results:**

Three main themes were identified. These were resource challenges, environmental/system challenges, and satisfaction with mental health services. Temeke health facility faced resource and environmental/system challenges, and there were mixed feelings on satisfaction with services. Funding and priority issues were found to mainly affect delivery of appropriate services to clients.

**Conclusion:**

Health facilities that provide mental health services in the community need to be well equipped with necessary resources to meet the vast needs of people they serve. Having a political will, improving the health systems governance for mental health, and priority setting, are necessary to address the challenges experienced in the delivery of mental health services.

## Background

Mental disorders are very common in almost all countries of the world, and are known to largely affect socioeconomic development and growth. The global DSV-IV disorders lifetime prevalence is estimated to be between 18.1% and 36.1%, with no significant difference between high and low and middle income countries. Mental disorders often have an early age of onset, and are associated with considerable societal costs [1, 2]. The social and economic impact of mental disability is diverse and far-reaching. Mental disorders inflict emotional as well as financial burden on individuals, families and society as a whole [3]. People with mental disorders are usually victims of homelessness, incarceration, lack of educational and income-generation opportunities, and human rights violation. This situation frequently leads individuals and families into poverty and hindrance of economic development at the national level [4, 5]. The impact of mental disorders on human life dictates the basis for designing and implementing effective strategies to address this problem.

There is a need to scale up treatment for mental disorders in low and middle income countries where most of the treatment gap exist [6]. This is thought to be potentially cost-effective and would result into many communities of the world to be more functional and productive [1]. For this reason, the World Health Organization (WHO), put down key recommendations for organizing mental health services through its Mental Health Policy and Service guidance package, to assist countries to improve the mental health of their populations [7]. The recommendations outline key 7 principles for organizing comprehensive mental health services in order to address various needs of people with mental disorders. These principles include accessibility, comprehensiveness, coordination and continuity of care, effectiveness, equity, respect for human rights, and coordination of specialized services with primary care and intersectoral collaboration.

However, low and middle income countries like Tanzania continue to face many challenges in the efforts to implement the WHO recommendations to improve mental health services. Such challenges include human resource constraints, inadequate training in mental health care, misplacement of mental health care professionals, lack of drugs, low priorities, lack of skilled care providers at primary health care level, problematic insurance coverage for mental disorders, and stigma [8–10]. Levels of public expenditures on mental health are very low in low and middle-income countries (less than US$ 2 per capita) [11]. These challenges are partly attributed to a lack of political will by governments in giving due priority to mental health [12]. As a result, a much lower proportion of low-income countries report having a policy, plan, and legislation on mental health than high-income countries. For example, while 92% of people in high income countries are covered by mental health legislation, only 36% are covered in low income countries [11]. To effectively implement programs aimed at meeting mental health needs, governments and health authorities of low and middle income countries must first issue clear policies articulating measures for the identification and treatment of patients with mental disorders [13].

This study was conducted in Dar es Salaam, the commercial and largest city in Tanzania. It is one of the fastest growing cities in sub-Saharan Africa and African continent, fueled by an influx of unemployed youth from upcountry and rural areas [14, 15]. According to the Population and Housing Census of 2012, the city has a growth rate of 5.6 which is about 2 times higher than the national growth rate (2.7). An increasing portion of the city’s population, including the newcomers, faces limited employment options and struggles under difficult living conditions [15]. There is also a high rate of illicit drug trafficking and drug abusers, according to Population and Housing Census of 2012. Such socioeconomic challenges are likely to cause mental health problems among the population [16, 17]. With this trend, mental health needs are rising, and district hospitals are being bombarded with a huge number of clients seeking mental health care. It is, therefore, imperative that the community and health facilities are well prepared to meet this demand. In view of this situation, this study was conducted to see how health facilities at the district level were prepared to meet the increasing mental health needs of the population, specifically, in Temeke district. Results from this study are useful in shedding some light on the status of mental health services, and in providing recommendations to improve services so that mental health needs are adequately met. Results can also be applied in other settings with similar context within and outside the country, especially in other low and middle income countries.

## Objectives

The major aim of this study was to determine how mental health services were organized and delivered at Temeke district hospital, in Dar es Salaam.

### Specific objectives

1. Determine the health care facility’s human resource for mental health situation
2. Explore experiences of patients and caregivers in receiving mental health care services at the health care facility
3. Describe the trend in the number of patients being seen at the mental health section of the facility
4. Describe factors affecting provision of mental health services
5. Describe managerial support provided to the mental health section

## Methods

### Study setting

This study was conducted in a randomly selected Temeke district, in Dar es Salaam. Dar es Salaam is a very fast growing and largest commercial city in Tanzania with the population of more than five million according to National Bureau of Statistics of 2016. Temeke is one of the three district in Dar es Salaam, with socio-economic characteristics likely to influence the occurrence of mental health problem/disorders.

### Study design

This was a cross sectional qualitative study that looked at how mental health services were organized and delivered.

### Study participants

The study participants included in this study were health care providers, patients with mental illness, caregivers of the mentally ill, and coordinators for mental health services at district and national levels. Informed consent was sought from all participants before being interviewed. However, for mentally ill individuals, informed consent was sought twice, first from their guardians, and then from patients themselves. Since interviews with patients were conducted within the health facility, it was convenient to provide further support to patients in case they needed any help related to their health. Patients were repeatedly reminded that it was at their own discretion to stop the interview and leave whenever they felt they needed to do so.

### Sampling procedure

A convenient sampling procedure was applied to get all participants. Care providers, Patients with mental illness, and caregivers who were present during data collection were included. Participants were mainly recruited on the clinic days (Mondays, Wednesdays, and Fridays) when the mentally ill patients were being seen. The inclusion criteria were; care providers who had worked in the mental health section for at least six months, patients who had a chronic mental illness and caregivers who had stayed with a mentally ill patient for at least six months. The exclusion criteria were; caregivers who had serious patients, patients who were mentally unstable, and care providers who were attending serious patients.

### Data collection

A total of 14 in-depth interviews were conducted, 7 with mental health care providers (including the district and national mental health coordinators), and 7 with patients attending the mental health clinics at the health facilities. Two focus group discussions (FGDs) were also conducted with 16 caregivers (each FGD comprising 8 caregivers). Areas covered in the interviews were human resource for mental health situation, experiences of patients in receiving mental health care services, number of patients being seen, managerial support provided to the mental health section, factors affecting provision of mental health services, and satisfaction with mental health services.

In-depth interviews and FGDs were conducted in Swahili, a common language to all participants, and all interviews were digitally audio-recorded. A semi-structured interview guide was used to interview participants. All interviews were conducted in the rooms located within the hospital premises. During FGDs, the moderator (author) led the discussion and kept the conversation flowing while the co-author was recording the interviews and taking some notes.

### Data analysis

**Fig. 1:**
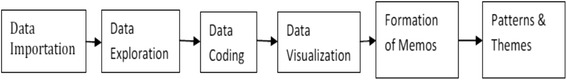
Steps followed in data analysis

All the audio data was transcribed verbatim. The content analysis method (Graneheim & Lundman, 2004) in accordance with qualitative analytical framework was used to analyse data. NVivo 10 software was used in organizing data and coding the text. The coded text was filtered and placed in similar contents that formed a family tree. The identified content of the text was entered into memos which were eventually manually organized into patterns and themes as shown below (Fig. 1):

## Results

### Characteristic of participants

The study comprised three groups of participants, namely, health care providers, caregivers, and patients. Health care providers included 2 Assistant Medical Officers (AMO), 3 Nurses, 1 Social Worker (SW), the District Mental Health Coordinator (DMHC), and the National Mental Health coordinator (NMHC). Among nurses who were interviewed, 1 was a nursing officer, 2 were senior nursing officers, and one was working as a social worker (See Table 1 below). The duration of providing mental health services among health care providers ranged from 3 to 18 years. Caregivers comprised both males and females. Patients who were included had schizophrenia, depression, and bipolar disorders.

**Table 1:** Characteristics of Participants

Group of participant	Total number
*Health care workers (HCW)*	
1. Nurses	3
2. Assistant medical officer	2
3. Mental Health coordinators	2
*Caregivers (CG)*	
1. Females	8
2. Males	8
Patients (PT)	7
Total	30

### Services offered

Mental health services being offered at Temeke health facility were limited to the outpatient clinic, counseling for drug abusers, and psychoeducation to relatives. When patients could not receive effective treatment from other places, including traditional healers, their last resort was at this hospital.

### Major themes

The analysis of FGDs and in-depth interviews revealed much information about the situation of mental health service delivery in Temeke district. Three major themes that emerged from this study were resource challenges, environmental/system challenges, and satisfaction with mental health services.

## Resource challenges

Resource challenges is one of the major themes that emerged that included inadequate mental health training, inadequate human resource for mental health, lack of psychiatric wards and associated services, limited space for service provision and worn out buildings, and inadequate diagnostic and treatment equipment.

- **Inadequate Mental Health Training**

Responses from mental health care providers and national coordinators indicated challenges with the capacity to provide professional mental health care. Most of care providers had no prior training in mental health, but were providing care to patients based on experience gained since they begun working in the mental health section. Only two nurses in the section had prior training as explained by a nurse:

“*I didn’t get any training…. I am a general counselor, so I have been using the counseling skills I had before in the cases like suicide, drinking poison, I just use my experience.”* (Social Worker)

*“To be honest, I studied for two years and qualified as a psychiatric nurse at Mirembe (School of Nursing). It’s only the two, a few of us (in the section,) who have gone through that.”* (Nurse)

The national coordinator reiterated the gap in prior training and was specifically concerned about the effectiveness of mental health services as expressed below:

*“The second challenge is about the effectiveness; the effectiveness of care provision is decreasing because, as I have said, that challenge of specialization for nurses, those nurses working in these (psychiatric) wards most of them do not have that specialty. Therefore the effectiveness of services provided decreases.”* (Nurse-midwife)

Regardless of lack of prior training in mental health for most health care providers, on-job or refresher training, if at all, happened rarely due to lack of funds and priority problems as expressed by the District Coordinator and social worker:

*“… If there would be training, especially on mental health issues. But these capacity building trainings are not there and there is no money, no budget, so if stakeholders (to help) could be available we would be grateful.”* (District Mental Health Coordinator)

*“So who will provide (on-job training) when no decision makers knows (laughs). May be it (on-job training) should come from outside people, if it’s here, they will not do it for mental health, they will go and teach other things although you are from the mental health section (laughs).”* (Social Worker)

(b). **Inadequate Human Resource for Mental Health**

Apart from receiving inadequate training in mental health, care providers were very small in number. The mental health section had no psychiatrist (there was one from the national hospital who was attached there temporarily). However, with this small mummer, care providers were seeing 800 to 900 patients in a month. They appreciated the availability of the attached psychiatrist as he helped with difficult cases, but complained that they were generally being overwhelmed by the increasing number of patients.

*“We only have one psychiatrist, and it’s not that we have a psychiatrist because we have borrowed him. He has been borrowed because he belongs to Muhimbili, that’s Dr James”* (not his real name).” (Social Worker)

*“As nurses we really are not enough, we may seem like we are enough because the place where we work is small, so when we are given a bigger place we will not be enough because patients are so many* …. even *doctors are also not enough because patients are so many.”* (Nurse-midwife)

The shortage of human resource for mental health was attributed to many factors including lack of interest in the program, retirement, and the government policy that shortened diploma and certificate nursing programs leading to exclusion of the mental health package for graduating nurses as described by the national and district coordinators:

*“It’s that in our municipal, we have a challenge of workers who would like to be trained in mental health, most do not like to be trained in mental health. Therefore you find that there are some centres that are run by nurses trained in mental health, but they are not many.”* (District Mental Health Coordinator)

(c). **Lack of Mental Health Wards and Associated Services**

Lack of inpatient mental health and associated services was verbalized by many health care providers as a big problem that made provision of care difficult as they were forced to refer patients who needed inpatient care to Muhimbili National Hospital.

*“We have been making noise so that we can be given at least a ward because this hospital has become big. They need to see us so that at least they build in one area, they need to prioritize and place wards for mental health since when we have a patient who needs hospitalization we refer to Muhimbili. So we do not have a place to hospitalize these patients. So that’s my cry for now.”* (District Mental Health Coordinator).

*“But for those of us who know our capacity, for example those who need inpatient management, those who need psychological psychotherapy, may be those who need occupational therapy; we do not have all these departments, so we only provide diagnosis and the management needed at that moment”.* (Assistant Medical Officer)

There were other challenges that health care providers experienced in the process of referring patients to Muhimbili National Hospital. Aggressive patients were not supposed to be sedated before they were referred, otherwise they could not be received at Muhimbili as they were required to be seen as they were before any medication were given. Sometimes it happened that when the patient was referred after being sedated, he was returned to the health facility. This caused a lot of problems in managing such patients, and care providers were forced to seek help from other people and sometimes to see patients right in the vehicles that brought them:

…. *We can provide care like medication to start with so that he (patient) becomes less aggressive. But at the referral place at Muhimbili they don’t like us sending a patient who has been treated, they say if we are to give a referral, then the patient should go as he is.”* (Nurse)

*“Before Dr. Simon (not a real name) became a psychiatrist he referred a patient, he was a general doctor, and the patient was very violent he could not even get into the ambulance because of aggressiveness. So they had to sedate him, but when he arrived at Muhimbili he was brought back. So that doctor had to stay with the patient at the reception until he woke up and referred him again. So it’s a challenge, we cannot send patients like that.”* (Assistant Medical Officer)

(d). **Limited Space for Service Provision and Worn Out Buildings**

The outpatient services being provided at the facility were faced with challenges of limited space and worn-out buildings. Limited space made it very difficult to provide services, privacy was compromised, and there was no security for health care providers who were seeing aggressive patients as explained below:

*“Another challenge is the service area as it is very small, it’s not enough .... The room has one door only, and as you know when those patients have relapsed they become very aggressive. So there should be another door in case you are overpowered so that you can at least take control, unlike having only one door where the patient can control you. This place is not good for security”.* (Nurse-midwife)

*“…We take history in the same room, make diagnosis right there, counselling right there, medication dispensation right there, if it’s an injection right there. So you can see that to work in an environment like this it is difficult … in the same room the nurses sits there, the doctor right there, we really work in a very difficult environment”.* (Assistant Medical Officer)

Patients were also concerned about the worn-out service area which was not renovated and the small waiting area which was located in the corridor where many people were passing. They had to, from time to time, stand up and allow people to pass as explained by a patient with schizophrenia:

*“but now the place is old, worn-out, very old and renovation has not been done … so we have to stand up and let people pass … the waiting area is used as passage for people … the psychiatric unit is very small, it’s very small”.* (Schizophrenia patient)

(e). **Inadequate Diagnostic and Treatment Equipment**

There was lack of diagnostic and treatment equipment at the health facilities which was said to be affecting provision of mental health services as stated by the national coordinator and a nurse:

*“The availability of diagnostic equipment like EEG for mentally ill patients is still a challenge. Still there is another diagnostic tool that causes short circuit called ECT which is still a challenge in our hospitals ... So those are the challenges I am talking about that we are facing …”* (Nurse-midwife)

*“With equipment/tools in the section, we only have BP machine and thermometer, and that stethoscope, that’s all.”* (Nurse)

## Environmental/system challenges

The second theme that strongly emerged was the Environmental/system challenges which included misallocation of trained human resource for mental health, use of offensive language by care providers, long waiting and short consultation time, increasing number of patients, and care providers’ lack of support from higher authorities.

- **Misallocation of Trained Human Resource for Mental Health**

Health care providers acknowledged that there were nurses who were trained in mental health but were working in other sections or departments of the hospital. They were placed there because they were considered very hardworking and efforts to relocate them back to mental health section seemed as a misuse of resources as explained by a nurse midwife and assistant medical officer:

*“But many psychiatric nurses, most of those who have been trained are not in the section (mental health), they have been placed in other sections because they are hardworking”.* (Social Worker)

*I have already told you that here there are psychiatric nurses, but they are not allocated to us … I read from reports everyday for example we have 900 patients per month and people are shocked with this number. But when it comes to requesting for staff, to get a psychiatric nurse from (other) wards and bring them here they think it’s like losing resource.”* (Assistant Medical Officer)

(b). **Use of Offensive Language**

Caregivers commented on the language that sometimes was used by care providers and termed it improper or offensive. Services delivery in the absence of proper language to clients was considered not enough no matter how good it was. Caregivers were of the opinion that proper communication with patients is an integral part of treatment as explained by a care giver:

*“But sometimes services are not very friendly, some harsh languages are often used which is not good. We can be given medication, but medication by itself is not enough, words of comfort can heal a person better than that a pill*. ... *So we are really not satisfied with the language that is used though it depends on the condition of the patient. The language of our care providers is not very good.”* (Caregiver)

(c). **Long Waiting and Short Consultation Time**

Patients and caregivers complained about unnecessary long waiting time at the facility which was attributed to care providers making stories, reporting late, and presence of many clinical students. Patients were also not satisfied with the time spent with care providers as they claimed it was too short:

*“there are complaints that doctors who see patients in there spend much time talking, telling stories…they talk to each other while patients pile up sitting outside there and waiting for service. But they (doctors) remain inside just talking, telling stories, laughing. This situation makes patient complain …but the time spent with the doctor is little.”* (Schizophrenia patient)

(d). **Increasing Number of Patients**

The other important challenge that care providers were concerned about was the increasing number of patients in the section and nationally, with Dar es Salaam being the leading region. Up to 900 patients were seen at the section in a month, and on a busy clinic day they could see even up to 100 patients. Given the number of care providers in the section, the number of patients being seen was just too big as stated below:

*“First, the problem or condition of mental illness here in this country has been increasing because with the statistics that we have it has been increasing every year. If we start with the statistics or year 2014/2015 the problem seems to have increased 3 times compared to the previous statistics, from 300,000 to 900,000 annually. ... Dar es Salaam is still the leading region.”* (Nurse-midwife)

*“With this service (mental health), people are now aware and I can see even up to 900 or 1,000 patients per month … days are different, there are those hot clinic days you can see 80 up to 100 patients.”* (Assistant Medical Officer)

(e). **Support from Management and Higher Authorities**

Care providers were asked about the support they received from higher authorities in the course of executing their daily duties in the section. Responses revealed that at all levels of the care delivery system, mental health was not given due weight or importance it deserved, and as a result it suffered small budgets and priority issues. Care providers were not happy with the support they received from the hospital management on issues that affected delivery of services including the allocation of care providers to the mental health section as mentioned by the assistant medical officer:

*“The psychiatric department is left far behind; people don’t see the importance of sending workers. … This is the responsibility of the matron, the matron allocates people to, I don’t know. IPD, OPD manager, is the one who says this one should go to a certain place and this one to a certain place.”* (Assistant Medical Officer)

Lack of support from higher authorities was also acknowledged even at district and national levels.

*“But when it comes to budget issues, they have their own priorities and they have not given priority to those in mental health. ... You find them rushing to maternal and child health and other treatment equipment. ...”* (District Mental Health Coordinator)

## Satisfaction with the mental health services

The third theme that emerged was participants’ satisfaction with the mental health services that were being offered at the facility. Health care providers, caregivers, and patients, as people directly involved in the care provided, were asked about their general impression or satisfaction with the mental health services. They gave differing responses as some were positive and satisfied while others were not.

Various reasons were given for the positive views and satisfaction with mental health services. Care providers viewed services as better because they were able to stabilize the condition of their patients. Patients who were positive about the services appreciated how they were received and treated, and how their conditions changed over time. Satisfied caregivers appreciated the way they were received, counseled, and the humility shown by health care providers:

*“…so I am very happy because I take a person from confusion state to come to his/her normal state. Therefore, I personally get very comforted, I feel peaceful because I take mentally ill patients from a very bad condition and get them back to their conditions, and there are those who get cured completely. For those who don’t get cured you at least get them to some place.”* (Assistant Medical Officer)

*“…really the services are good, doctors treat us just well, when we come they receive us well, they ask as questions, generally the services are good.* ... *It’s (service) not bad because I could not eat and sleep, but when I started to use the medication I started to eat and sleep”*. (Bipolar patient)

*“The service is not bad because when you come here you are received well and counseled well, and the doctors are humble.”* (Caregiver)

With regard to negative views and dissatisfaction with services, participants gave various reasons. Care providers were concerned about insufficient medication which was a major factor affecting services and causing patients’ dissatisfaction. Patients attributed their dissatisfaction to service environment and lack of medication, while caregivers were concerned about the unchanging patient’s condition:

*“...but there are times when it becomes difficult, like right now we have a challenge of lack of medication...some (patients) are satisfied, others are complaining a lot and it’s because of this situation of not getting medication, they are complaining.”* (Nurse-midwife)

*“The services are not yet (good) when I compare with those I was receiving when I went to a private hospital, and the environment there is better than here”*. (Schizophrenia patient)

*“The other challenge is, I don’t know about services here, I am not satisfied because sometimes I see him coming by himself (the patient) to the clinic and going back, I don’t know what happens with the doctor, I am not satisfied.”* (Caregiver)

### Plans and efforts to improve mental health services

With all the challenges explained in this study, mental health authorities at district and national level had some plans in place to improve the situation. However, it was not clear how realistic these plans were, given the challenges they also acknowledged. They talked about various strategies like bringing back essential drug kits, integrating mental health in primary health care, revising diploma nursing training programs to strengthen mental health, and sending people to specialize in mental health:

*“The first strategy is that we are bringing back the essential drug kits, the second strategy which is parallel with that, is that we have been working on integrating mental health services into the primary health care … the third strategy is that, as a section, we have started meeting with our colleagues from the Training Unit, Nursing Director, and other stakeholders to see how we can bring back the mental health training for diploma…”* (Nurse-midwife)

*“There are efforts made to get a doctor who has gone for mental health degree, he is called Peter (not his real name) and went for studies to Lushoto, we expect that when he comes back he will help us. Another one is expected to graduate soon also from Lushoto, may be when these two come they may help us with this problem. But convincing nurses and doctors to specialize in mental health, it’s a little bit difficult.”* (District Mental Health Coordinator)

When asked on what needed to be done to improve mental health services at the health facility, patients, caregivers, and care providers gave many suggestions including patients being exempted from treatment costs, expanding the service area, having more competent health care providers, having more days or time for service, more and advanced investigations being done for patients, prioritizing and strengthening mental health services, and to reduce waiting time. Health care providers, particularly, needed to be given some risk allowance and motivation for the work they did.

## Discussion

This study reveals limited mental health services which were being offered at Temeke hospital including outpatient clinic, counselling for drug abusers, and psycho-education. This corroborates findings from low income countries which show that many health service organization requirements for mental health care are absent [10, 18]. Having limited mental health services makes it very difficult to meet the diverse needs of patients who visit health facilities for treatment and care. It was acknowledged that patients sought informal mental health support from various places but their last resort was at the hospital. When patients were not able to get service from elsewhere, including from traditional healers, they believed they would get it at the hospital. Seeking informal mental health care is a known practice in sub-Saharan Africa and people who are often sought include traditional healers and spiritual leaders [19]. The majority of clients use both the traditional and biomedical models of treatment [20]. The behaviour of seeking treatment is embedded within contextual cultural milieu, reflecting community beliefs, experiences, religion, and spirituality [21]. However, it is not part of the aim of this study to discuss in details mental health treatment seeking behaviour of participants.

Three major themes which emerged from this study are discussed. These are resource challenges, environmental/system challenges, and satisfaction with mental health services.

### Resource challenges

This study reveals mental health resource challenges that Temeke health facility was facing, including inadequate mental health training, inadequate human resource for mental health, lack of psychiatric wards and associated services, limited space for service provision and worn out buildings, and inadequate diagnostic and treatment equipment. This can be explained by what is known about low and middle income countries where mental health resources are very scarce and investment is minimal. Mental disorders do not attract global health policy attention and expenditures are very low due to lack of political will and prioritization [11, 12]. This situation reiterates the importance of mental health leadership and governance in the country and the need for the government to play its role in guiding and overseeing the mental health system, as emphasized by WHO. This means strategic policy and legislative framework have to be in place, and if they are, then effective oversight and accountability mechanisms have to be established [22]. Mental health systems strengthening in low and middle income countries is strongly being advocated for, with focus on capacity-building of researchers, policy makers, and planners [23]. If these actions are not taken, then mental health services cannot be expected to improve in these countries, and the challenges experienced will keep on existing. In the following paragraphs specific resource challenges that were revealed in this study are discussed in detail.

One of the resource challenges experienced at Temeke health facility was inadequate training of human resource for mental health. The majority of mental health care providers had no prior training in mental health but were providing care to patients based on personal experience, which in a way compromised the capacity to provide quality care to clients. Moreover, on-job or refresher training happened rarely due to lack of funds and priority problems. Inadequate training in mental health and lack of refresher courses have been reported in other low income countries [8]. This situation compromises care, and makes care providers feel incompetent [22, 24]. Regular refresher training is critical in ensuring appropriate care for people with mental disorders. When it’s absent it raises the possibility that patients in these countries are receiving substandard care, as they are not benefiting from the latest advances and knowledge in the field [10, 25].

Another resource challenge that strongly came up in this study was inadequate human resource for mental health. The facility had very few mental health care providers compared to the number of patients being seeing. They had no psychiatrist, but used to borrow one from Muhimbili National Hospital, who was attached there on a temporary basis. The shortage of human resource for mental health was attributed to lack of interest in the program, retirement, and government policy that shortened diploma and certificate nursing programs leading to exclusion of the mental health package for graduating nurses. This corroborates with findings from other studies which show that inadequate or lack of human resource for mental health is a common problem in low and middle income countries [6, 10, 19, 22, 25, 26]. Known reasons for this shortage from other studies include little time devoted to mental health within professional training programmes, poor working conditions, low status of the profession, which mean that few people enter the mental health professions, and among a few who graduate some are brain drained for greener pasture in other countries with better working conditions [6, 25]. The shortage contributes to having the majority of people with mental disorders not receiving evidence-based care, leading to chronicity, suffering, and increased costs of care [12]. Many strategies have been proposed to address this problem including task shifting and prioritizing organization and ways of delivering community-based mental health services [27].

Apart from human resource challenges, the facility was facing other non-human resource challenges, one of which was lack of psychiatric wards and associated services such as psychotherapy and occupational therapy. Lack of wards led to failure to admit patients who needed inpatient care and being forced to refer them to Muhimbili National Hospital. One would wonder why at such a level of health facility psychiatric wards would be missing. However, lack of such inpatient facilities have been reported in other low and middle income countries [22, 25, 28]. Lack of other services associated with mental health such as psychotherapy and occupational therapy have also been reported and attributed to lack of skilled human resources and the acceptability of treatments across cultures [29]. Inpatient psychiatric facilities are important in serving a few patients in acute conditions who need treatment and close observation [22]. However, the issue of providing referral to all patients with acute condition does not support the global efforts for decentralizing and integrating mental health services in the primary health care, but it actually contributes to overcrowding of psychiatric wards [30].

Another non-human resource challenge that was being experienced at Temeke health facility was limited space for mental health services and worn-out buildings, which resulted into lack of waiting area and compromised patient privacy and the security of care providers especially when they were attending patients. Such a situation just confirms a well known fact that mental health services have been widely neglected in low and middle income countries compared to other services [27, 31]. In South Africa for example, barriers to the financing and development of mental health services resulted in psychiatric hospitals remaining outdated, falling into despair, and often unfit for human use [26, 32]. Although evidence is not adequate to support the role of the physical environment of mental health services on patient outcomes, research has suggested that, indirectly, the physical environment may influence mental health by altering psychosocial processes with known mental health sequelae. Personal control, socially supportive relationships, and restoration from stress and fatigue are all affected by properties of the built environment [33]. Therefore it might be helpful to consider the patients’ personal characteristics when designing the physical environment for them [34]. Governments in low and middle income countries need to make deliberate efforts to rescue this situation [27].

Inadequate diagnostic and treatment equipment was also among the non-human resource challenge that affected mental health service provision at Temeke hospital. Participants mentioned basic diagnostic equipment such as blood pressure machine, thermometer, and stethoscope which were not available for assessing the physical condition of patients. Since co-morbidity in psychiatric disorders is very common [35, 36], providing holistic care to patients is required [37]. This signifies that such basic diagnostic equipment should be available for assessing patients and disposing them accordingly. Lack of treatment equipment such as electroconvulsive therapy, which is still accepted in Tanzania, just echoes the treatment gap for mental disorders in low and middle income countries [6] and the need to reduce it [38].

### Environmental/system challenges

A number of environmental/system challenges were reported in this study including the misallocation of trained human resource for mental health, use of improper language by health care providers, patient long waiting and short consultation time, increasing number of patients being seen, and lack of support from the hospital higher authorities.

The scarcity of human resource for mental health has been discussed previously as part of the resource challenges faced at the health facility. However, this situation was complicated by the misallocation of trained human resource for mental health, as one of the system challenges. It was reported that a number of nurses who had prior training in mental health had not been allocated in the mental health section/department but were working in other areas of the hospital. It was difficult to re-allocate them back to mental health section for various reasons including their leaders being reluctant to lose them as hard-workers. Issues of misallocation of human resource for mental health have been reported in low and middle income countries such as Zambia [8, 26]. Given the already existing huge shortage of human resource for mental health and the burden of care in low and middle income countries [10, 26], misallocation of psychiatric nurses should be avoided by all means so that this scarce resource is made available for mental health services. It is also equally important to establish research evidence on why some health care providers do not prefer to work in psychiatric settings regardless of receiving prior training.

Another system challenge that came up in this study was the use of improper language by health care providers. Some caregivers and patients reported being offended by the language used by some care providers. Unfortunately, there is a dearth of documented evidence of care providers using offensive language towards clients. However, it has been reported that in some cases patients are either physically or psychologically mistreated by doctors and nurses. Such mistreatments may include verbal abuse, not being given appropriate information, and socio-economic discrimination. Care providers’ own unethical behaviors are attributed to this malpractice, and this calls for internalization of professional and ethical roles so that they provide safe and ethically sound care [39].

Patient long waiting and short consultation time was another system challenge verbalized mainly by patients. Patients attributed this problem to care providers making stories, presence of many clinical students in the service room, and health care providers reporting to work late. Time spent with care providers was considered too short by patients. Other studies have reported long waiting time caused by delays in moving from one service point to another, attitudes of staff, and low staff-patient ratio [40, 41]. May be, low staff-patient ratio could explain the delay in seeing patients as a result of increasing number of patients as discussed in the following paragraph. With consultation time, sometimes patients have intense sense of time pressure and a self imposed rationing of time that actually affect quality of care. That’s why care providers need to be aware of the anxieties that lead to patients fear of using up their time during consultation [42]. In this way, patients’ complaints about short consultation time could be reduced.

Yet another system challenge care providers and mental health coordinators were concerned about was the increasing number of patients, with Dar es Salaam being the leading region in Tanzania. This corresponds with what has been happening globally, where mental disorders have been on the increase, largely due to changes in population growth and aging. Population growth and a changing age profile are producing a shift in the disease burden from communicable to non-communicable diseases and from years of life lost (YLLs) to years lived with disability (YLDs), a transition contributing to a rise in the of burden mental disorders in developing regions [43]. Given the state of mental health services in these regions, and the shortage of human resource [22, 28], the situation is expected to get worse in the absence of proper interventions. The alarming and rising health, economic, and social burdens associated with mental health disorders call for evidence based interventions that would alleviate suffering and curtail the associated economic and social consequences of unmet needs [27].

We conclude the discussion on system challenges with lack of support from the hospital higher authorities. Care providers in this study were not happy with the support they received from the hospital management and higher authorities on issues that affected delivery of mental health services including supervision and allocation of care providers in the mental health section. They voiced out that the mental health section was not given due weight or importance, thus it suffered small budgets and priority issues, which was a demoralizing situation. It is known that lack of mental health coordination and supervision may negatively affect provision of mental health services [44]. Supportive managers and well designed organisational procedures and structures in the health facility are crucial factors for maintaining good staff morale. Unsupportive environment may lead to insufficient staffing levels and high risk of violence and could be devastating to staff morale [45]. Therefore there is a need for health managers and higher authorities in health facilities to create and maintain a supportive environment that would motivate care providers to work comfortably, thus contribute to realization of organizational goals.

### Satisfaction with mental health services

Satisfaction with mental health services is the third and last theme discussed here. Participants had both positive and negative views about mental health services offered at the health facility. Satisfaction with services was related to how clients were received, treated, and improvement in patient condition over time. Dissatisfaction with services was attributed to poor service environment, lack of medication, and unchanging patient condition. Although similar studies in low income countries could not be easily found, mixed feelings about satisfaction with mental health services have been reported in other parts of the world such as southern Sweden, Norway, and USA [46–48]. Patient satisfaction in psychiatry is a complex issue which has various influencing factors such as age, residence, type of disorder, duration of illness, and social functioning [49–51]. Satisfaction with services is also related to fulfilment of patient expectations, as well as a perception of fairness [52]. Therefore, integrating patients to their own treatment plan, regular service evaluation, and improving communication, are important to improve satisfaction.

## Limitation

This study included care providers working in the mental health section, patients, caregiver, and district and national mental health coordinators. Higher authorities within the Temeke hospital were not included. May be, doing so might have altered the results of this study because of the role they play in the delivery of health services such as allocation of human and material resource and moral support.

## Conclusion

This study illuminates mental health service organization and delivery at Temeke hospital and the challenges experienced. With all its dedication to care for the needs clients, the health facility faced resource challenges, environmental/system challenges, and participants had mixed feelings about satisfaction with mental health services. Funding and priority issues mainly affected delivery of appropriate services to clients. Therefore we recommend that mental health services in the community should be well equipped with necessary resources to meet the vast needs of the people. Capacity building for human resource for mental health in terms of basic training and refresher courses needs to be emphasized so that more health care providers are produced and kept abreast with latest advances in knowledge and skills. Equally important, task shifting policy needs to be implemented as one of the strategies to reach a larger population that does not receive mental health care. There is a need to improve health systems governance so that it’s more responsive to the increasing mental health needs. Finally, there is a need to strongly advocate for prioritization and better budgets for mental health services. Government leaders and other stakeholders, including development partners, have a big role to play in ensuring that improvement in mental health services is realized in the country.
